# Combining Nanocarrier-Assisted Delivery of Molecules and Radiotherapy

**DOI:** 10.3390/pharmaceutics14010105

**Published:** 2022-01-03

**Authors:** Eliza Rocha Gomes, Marina Santiago Franco

**Affiliations:** 1 Department of Pharmaceutical Products, Faculty of Pharmacy, Universidade Federal de Minas Gerais, Belo Horizonte 31270-901, Brazil; elizarochagomes@gmail.com; 2 Department of Radiation Sciences (DRS), Institute of Radiation Medicine (IRM), 85764 München, Germany

**Keywords:** nanocarriers, nanosystems, chemotherapy, radiotherapy, radiosensitizer, abscopal effect, hypoxia, synergism, cancer

## Abstract

Cancer is responsible for a significant proportion of death all over the world. Therefore, strategies to improve its treatment are highly desired. The use of nanocarriers to deliver anticancer treatments has been extensively investigated and improved since the approval of the first liposomal formulation for cancer treatment in 1995. Radiotherapy (RT) is present in the disease management strategy of around 50% of cancer patients. In the present review, we bring the state-of-the-art information on the combination of nanocarrier-assisted delivery of molecules and RT. We start with formulations designed to encapsulate single or multiple molecules that, once delivered to the tumor site, act directly on the cells to improve the effects of RT. Then, we describe formulations designed to modulate the tumor microenvironment by delivering oxygen or to boost the abscopal effect. Finally, we present how RT can be employed to trigger molecule delivery from nanocarriers or to modulate the EPR effect.

## 1. Introduction

Cancer is recognized as a leading cause of death all over the world. The disease is a barrier to increasing life expectancy, and its incidence and mortality keeps growing rapidly throughout the world. According to GLOBOCAN 2020, there were 19.3 million new cancer cases and around 10 million deaths from it in 2020 [1]. Currently, the main strategies used in cancer management are surgery, chemotherapy, and radiotherapy (RT). Chemotherapy acts not only in tumor cells but also in normal tissues, leading to systemic side effects that limit the doses that can be administered to the patients. One strategy to reduce the toxicity to normal tissues, thus enhancing the therapeutic index of chemotherapeutics, is the use of nanocarriers [2]. Nanosized formulations rely on their ability to passively accumulate in the tumor due to the enhanced permeability and retention (EPR) effect, discovered more than 30 years ago. Briefly, the EPR effect consists of nanocarriers taking advantage of the defective vascular architecture and poor lymphatic drainage in the tumoral area, to passively accumulate in this region [3,4]. This effect has been validated in both experimental animal models and different human tumors [3]. Since the approval of the first nanocarrier (Doxil^®^, liposomal doxorubicin) by the FDA in 1995, these formulations have been extensively researched and improved [5]. These improvements consist of different strategies. Active targeting to the tumor tissue is one of them. By decorating the surface of the nanocarriers with ligands directed to receptors known to be overexpressed in the tumor cells, it is possible to significantly increase drug delivery to the tumor as compared to passive targeting only [4,6]. Enhancing drug release kinetics at the tumor is also critical for the antitumoral effects. Therefore, nanocarriers designed to release their contents only when exposed to a trigger stimulus, either endogenous or exogenous, lead to superior anticancer effects [7]. The tumor microenvironment (TME) consists of different types of cells and many molecules released by tumor, stromal, and immune cells. It plays a role in tumor growth, differentiation, invasion, epigenetics, and immune evasion [8,9]. The complexity of the TME makes it an important target for cancer therapy with nanocarriers, which can carry and deliver multiple drugs with different targets [9,10]. The possibility to co-encapsulate molecules in nanocarriers allows for theranostic applications [11,12] as well as combination chemotherapy [13]. For combination chemotherapy, nanocarriers have been further finely designed to deliver specific synergistic ratios of drugs, significantly enhancing the antitumor activity [14,15]. 

The combination of different strategies to fight cancer will shape the future of cancer management. Understanding the mechanisms of strategies that can work together will lead to the greatest anticancer effects [2]. One strategy that is leading to good results is the combining nanocarrier-assisted delivery of molecules and RT. Commercialized nanocarriers such as Caelyx^®^ (liposomal doxorubicin) and Abraxane^®^ (albumin nanoparticles of paclitaxel) have already been combined to RT in clinical trials, showing to be a promising and safe treatment strategy [16,17,18]. These formulations, however, were not designed specifically with the purpose to be combined to RT. 

**Figure 1:**
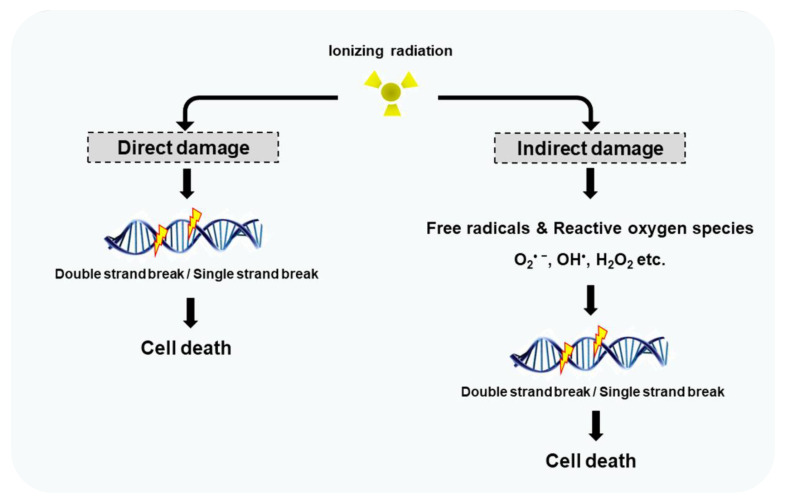
Ionizing radiation damages the DNA by direct and indirect effects. Direct damages arises from direct interaction between radiation and cellular DNA. Indirect DNA damage is caused by free radicals prevenient of the radiolysis of water molecules present in the cells. Reproduced from Hur and Yoon, MDPI, 2017 [26].

RT deposits energy in the cells damaging their genetic material, thus hindering their ability to divide and proliferate. The damage to the cells occurs in a direct or indirect manner as illustrated in Figure 1. The direct action comprises approximately 50% of the damage to DNA. The direct-type effect consists of two different events. The first arises from energy deposited in the DNA itself, so that sites of electron loss (radical cations), electron gain (radical anions), and excitations (minor role) are created through ionizations. The second event consists of quasidirect effects, arising from the DNA solvation shell. When the solvation shell is ionized, radical cations and ejected electrons are rapidly transferred to DNA [19,20]. The indirect action consists of the radiolysis of water molecules present in the cells producing free radicals such as superoxide, hydrogen peroxide, and hydroxyl radical. These reactive oxygen species (ROS) interact with cellular molecules, such as DNA, lipids, and proteins [20,21]. ROS-mediated cell death is mainly caused by clustered DNA strand lesions, which are difficult to repair. The membranes and organelles of cells are also believed to be major targets of ROS. It acts by peroxidizing the membrane lipids, leading to structural and functional impairment, contributing to cell cycle arrest and apoptosis. Apoptosis can also arise from ROS-altered cellular homeostasis and ROS-modified signaling pathways [22]. Mitochondrial dysfunction due to ROS-mediated mitDNA damage leads to a sustained increase in endogenous ROS production, which culminates in more cell damage [23]. ROS are also related to early and late effects of RT, such as the bystander effect, field effect, inflammation, and fibrosis [24]. The direct and indirect disruption of the DNA molecular structure can lead to single-strand breaks (SSBs), base oxidation, apurinic, or apyrimidinic sites, and double-strand breaks (DSBs, the most important DNA damage) which culminate cell damage or death [25]. 

**Figure 2:**
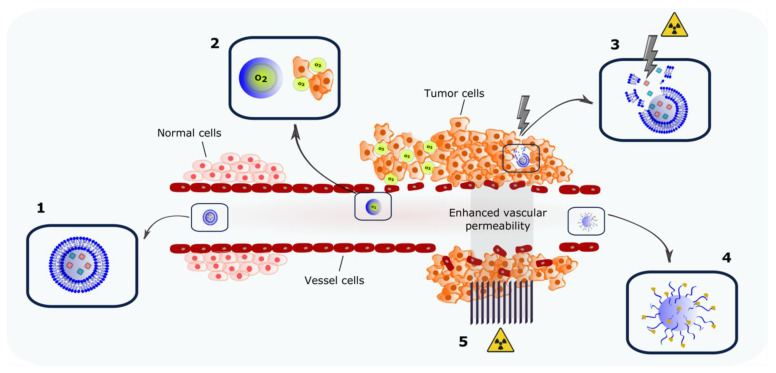
Strategies for combining nanocarrier-assisted delivery of molecules and radiotherapy. Encapsulation of single or multiple radiosensitizing agents in a nanocarrier (**1**); delivery of oxygen to diminish tumor hypoxia (**2**); radiation as exogenous triggering stimulus for in situ compound release (**3**); antigen-capturing nanocarriers to boost the abscopal effect; and (**4**) induction of transient enhanced vascular permeability by micro- and mini-beam irradiation modulating the EPR effect (**5**).

It is estimated that around 50% of cancer patients receive RT in their disease management. RT can be used as an isolated radical treatment or combined with surgery or systemic therapy in the curative setting. For patients with locally advanced or disseminated cancer, it is used to provide some symptom relief [27]. During RT, radiation doses that can be delivered to the patient are limited by normal tissue tolerance. Over the past few decades, thanks to engineering and computing, radiation instrumentation has strongly evolved. This allows an improved therapeutic ratio as radiation is delivered to the tumor with great precision, thereby minimizing normal tissue exposure, leading to higher cure rates. Additionally, radiobiology knowledge of tumor radiation sensitivity and resistance combined to normal tissue toxicity has improved RT outcome. The combined treatment of radiation and systemic drugs affects the radiobiological mechanisms in tumor and normal cells and is used in a large proportion of patients. Despite the good tumor control observed in many patients nowadays, some tumor types remain insensitive to RT or recur shortly after the treatment, indicating that there is still room for improvement [28,29,30]. The promising combination of nanocarriers and RT is the focus of the present review. Herein, we present how nanocarriers can be designed to modulate the effects of RT by delivering molecules that act either directly on the tumor cells or on TME. RT, in turn, can be used either as exogenous nanocarrier-trigger stimulus or to modulate the EPR effect. A summary of these strategies is depicted in Figure 2.

## 2. Nanocarriers Encapsulating Radiosensitizers

A range of compounds that influence, for example, DNA repair (especially double-strand break repair), act as radiosensitizers by increasing damage to the irradiated cells, leading to increased cell death [30]. However, these compounds present side effects that sometimes hamper their use [31]. Effective radiosensitizers should have less effect on normal tissues [21]. In this scenario, nanocarriers play an important role in directing the radiosensitizer to the tumor cells, sparing the normal tissue of the additional damage caused by irradiation. 

### 2.1. Chemotherapeutic Drugs That Act as Radiosensitizers

Combinations of conventional chemotherapeutic drugs with RT led to most of the significant advances in cancer treatment in the last decades. Therefore, combinations of chemotherapy and RT are today the standard of care for many patients with solid tumors [32,33].

#### 2.1.1. Cisplatin

Platinum analogs such as cisplatin (CDDP) are well-known radiosensitizers that have been widely used clinically in combination with RT for cancer treatment. Different potential mechanisms associated with radiation potentiation have been reported. Some of these mechanisms consist of adduct formation and DNA damage repair inhibition, an increase in cellular platinum uptake induced by radiation, synergistic effect due to cell cycle disruption, and enhanced formation of platinum intermediates when radiation-induced free radicals are present [32,33]. However, the use of CDDP is limited due to its severe nephrotoxicity [34], which can be overcome by its encapsulation in nanocarriers. Zhang et al. developed liposomes encapsulating CDDP (L-CDDP) and evaluated its antitumor efficacy in combination with RT (6 Gy) in a mouse model with human lung adenocarcinoma A549 tumor. L-CDDP plus irradiation led to a higher tumor growth suppression compared to CDDP plus irradiation and irradiation alone. The tumor growth delay (TGD) values were 11.95, 3.27, and 1.83 days, respectively. Sensitizer enhancement ratio (SER) values, when drugs were administrated 72 h before radiation, were 3.21 for CDDP and 4.92 for L-CDDP, confirming the effective role of L-CDDP in directing the radiosensitizer to the tumor [35]. More details about all formulations presented in this review can be found on Table 1.

Jung et al. [36] prepared liposomes modified with epidermal growth factor receptor (EGFR) antibodies encapsulating CDDP (EGFR:L-CDDP). A colony formation assay (CFA) was performed on A549 cells treated with the free drug, actively and nonactively targeted formulations combined to irradiation (0, 2, 5, or 10 Gy) 2 h later. This assay consists of an in vitro cell survival assay which assesses the ability of a single cell to grow into a colony, defined as a group of at least 50 cells. It is the gold-standard method in radiobiology to determine cell reproductive death after exposure to ionizing radiation [37]. This preliminary study indicated that both liposomes lead to enhanced radiosensitivity, with the actively targeted formulation being slightly more potent. The antitumor efficacy was evaluated in animals bearing A549 tumors. On the final date of the experiment, the change of tumor growth was compared between treated group and control group (T/C). The percentual changes of tumor growth were calculated as: T/C (%) = [(change in tumor growth for treated group)/(change in tumor growth for control group)] × 100. The T/C (%) were 53.1 for free CDDP, 61.3 for L-CDDP, and 46.8 for EGFR:L-CDDP. After the combination of drugs with irradiation (5 Gy), these values were 40.7, 32.2, and 20.7, for free CDDP, L-CDDP, and EGFR:L-CDDP, respectively, while it was 51.8, for the group treated with irradiation alone (5 Gy). These results reveal a higher efficacy of EGFR:L-CDDP either alone or combined to irradiation [36].

**Table 1:** Nanocarriers designed to be used in combination with radiotherapy.

Formulation	Composition	Encapsulated Agent	Mean Diameter	Irradiation Dose (Gy) *	Reference
	**Nanocarriers Encapsulating Radiosensitizers**				
Liposome	HSPC:CHOL:DSPE-PEG2000	Cisplatin	~100 nm	6 Gy	[35]
Liposome	(DPPC):CHOL:ganglioside:DCP:DPPE) (35:40:15:5:5 molar ratio) and anti-EGFR antibodies	Cisplatin	247.9 nm	5 Gy	[36]
Liposome (*Promitil*^®^)	HSPC:CHOL:DSPE-PEG2000:MLP (60:30:5:5 molar ratio) HSPC:CHOL:DSPE-PEG2000:MLP (55:30:5:10 molar ratio)	Mitomycin C	98.61 nm	5 Gy	[38,39,40,41]
Liposome (*Myocet*^®^)	EPC:CHOL (55:34 molar ratio)	Doxorubicin	~160 nm	2 Gy	[42,43]
Liposome	DSPE-PEG2000:MDH:CHOL	Doxorubicin	169.4 nm	2 Gy	[44]
Micelles	PEG-PCL/P105	Doxorubicin	~20 nm	6 Gy	[45]
Nanoparticle	Precirol ATO, Pluronic F68, dimethyldioctadecyl-ammonium bromide	Curcumin	~300 nm	2 Gy to 9 Gy	[46]
Liposome	lecithin:CHOL:CUR (18:1:1 weight ratio)	Curcumin	114.9 nm	5 Gy	[47]
Liposome	DOPC:CHOL:DSPE-PEG2000	Cupric tirapazamine complex	160–180 nm	7 Gy or 10 Gy	[48]
Liposome	DPPC:MSPC:DSPE-PEG2000 (86:10:4 molar ratio)	Pimonidazole	~100 nm	4 Gy	[49]
Nanoparticle	H1 nanopolymer:Dbait	Dbait	170 nm	9 Gy	[50]
	**Co-delivery of Molecules: The Search for Synergism**				
Nanoparticle	PLGA-PEG	Cisplatin and Paclitaxel	82.9 nm	5 Gy	[51]
Nanoparticle	PLGA-PEG	Wortmannin and Cisplatin	80–200 nm	5 Gy	[52]
Nanoparticle	PLGA-PEG	Cisplatin and Etoposide	100 nm	5 Gy	[53]
Nanoparticle	PLGA-PEG:transferrin at a molar ratio of 1:3	Tetrahydrocurcumin and Doxorubicin	255.8 nm	3 Gy	[54]
Nanoparticle	angiopep-2:DSPE-PEG2000:DOTAP:PLGA	Temozolomide and Dbait	99.9 nm	3 Gy	[55]
Nanoparticle	H1 nanopolymer:Docetaxel:Dbait	Docetaxel and Dbait	117 nm	3 Gy	[56]
Nanoparticle	magnetic graphene oxide:FePt nanoparticles	Metronidazol and 5-fluorouracil	243 nm	2 Gy	[57]
Nanoparticle	(Poly-metronidazole)n:DSPE-PEG2000: lecitina:angiopep-2-DSPE-PEG-2000	Metronidazol and Doxorubicin	~80 nm	2 Gy	[58]
Liposome	DSPE-PEG2000: MDH: CHOL	Metronidazole and Dbait	127 nm	2 Gy	[59]
Nanoparticle	1,4-dicarboxybenzene (BDC): Hafnium (Hf):PEG	Talazoparib and Buparlisib	112 nm	4 Gy or 8 Gy	[60]
	**Nanocarriers Encapsulating Oxygen: Targeting Hypoxia**				
Nanoparticle	perfluorotributylamine (PFTBA)@albumin	Oxygen	150 nm	5 Gy	[61]
Nanodroplets	perfluoro-15-crown-5-ether (PFCE)@cisPt(IV)-Lip cisPt(IV)-Lip is prepared by mixing 2.5 mg cisPt(IV)-DSPE, 5 mg DPPC, 1.5 mg cholesterol and 4 mg DSPE-mPEG5k	Oxygen Cisplatin	~200 nm	6 Gy	[62]
Nanoparticle	PEG-Bi_2_Se_3_ @perfluorohexane	Oxygen	~35 nm	6 Gy	[63]
Nanoparticle decorated nanodroplets	TaOx@PFC-PEG	Oxygen	~150	6 Gy	[64]
Liposome	PFH@DSPE-PEG2000:CHOL:lecithin (3.79:4.28:24.65 weight ratio)	Oxygen	~100 nm	10 Gy	[65]
	**Nanocarriers Designed to Boost the Abscopal Effect**				
Nanoparticle	PLGA based NP coated with either amine polyethylene glycol; DOTAP or PEG-maleimide	-	<200 nm	-	[66]
Nanoparticle	Mesoporous silica nanoparticles functionalized with APTES	-	~100 nm	8 Gy	[67]
Nanoparticle	PEG-maleimide-mPEG-functionalized hollow mesoporous titanium dioxide (HTiO_2_)	IDOi (Indole-amine-2,3-dioxygenase inhibitor)	~50 nm	4 Gy	[68]
	**Radiation-Triggered Delivery Systems**				
Nanoparticle	DNA:AuNP	Doxorubicin	NA	5 Gy	[69]
Nanoparticle	bismuth nanoparticles functionalized with S-nitrosothiol	-	36 nm	5 Gy	[70]
Nanoparticle	Pegylated thioether-hybridized hollow mesoporous organosilica nanoparticles	tert-butyl hydroperoxide (TBHP) and iron pentacarbonyl (Fe(CO)5)	~50 nm	8 Gy	[71]
Liposome	DOTAP:DOPC (~1:1 weight ratio)	Doxorubicin	NA	4 Gy	[72]
Liposome	egg lecithin-80: DSPE-PEG2000 (60:9 *w/w*)	Hemoglobin and Doxorubicin	~140 nm	8 Gy	[73]

* Photon irradiation was used in all experiments. Abbreviations: (3-aminopropyl) triethoxysilane (APTES); cholesterol (CHOL); diacetyl phosphate (DCP); 1,2-dioleoyl-sn-glycero-3-phosphocholine (DOPC); 1,2-di-(9Z-octadecenoyl)-3-tri- methylammonium-propane (DOTAP); dipalmitoylphosphatidylcholine (DPPC); dipalmitoylphosphatidylethanolamine (DPPE); 1,2-distearoyl-sn-glycero-3-phosphoethanolamine-N-[amino(polyethylene glycol)-2000 (DSPE-PEG2000); folate–polyethylenimine600–cyclodextrin (H1 nanopolymer); hydrogenated Soy Phosphatidylcholine (HSPC); malate dehydrogenase (MDH); mitomycin and glycerol lipid (MMC lipid prodrug) (MLP); 1-stearoyl-2-hydroxy-sn-glycero-3-phosphocholine (MSPC); polyethylene glycol (PEG); polyethylene glycol-polycaprolactone/pluronic (PEG-PCL/P105); and poly(lactic-co-glycolic acid) (PLGA).

#### 2.1.2. Mitomycin

Mitomycin C (MMC) is a DNA crosslinking agent considered to be a potent chemotherapeutic drug and radiosensitizer. It forms DNA adducts compromising the repair of radiation-induced DNA breaks by cells. MMC is attractive as a radiosensitizer as it may target hypoxic cell populations in detriment to oxygenated cells; however, its use is still associate with significant toxicity such as pulmonary fibrosis, hemolytic uremic syndrome, and damage to bone marrow and other tissues [38,39,40]. Thus, Gabizon et al. developed a lipidic prodrug consisting of MMC linked to 2,3-distearoyloxy-propane-1-dithio-4′-benzyloxycarbonyl, abbreviate as MLP and formulated into liposomes, known as Promitil^®^ [40,41]. A phase 1A clinical study with Promitil^®^ showed that toxicity was substantially reduced. Currently, Promitil^®^ is in a phase 1B clinical study [38]. In vitro cytotoxicity of free MMC and Promitil^®^ against colorectal cancer cell lines HT-29 and SW480 was evaluated, and both cell lines showed a dose-dependent response to the treatments. When HT-29 and SW480 cells were irradiated (doses ranging from 0 to 8 Gy) after drug treatments (10 nM MMC), radiation survival curves demonstrated that both Promitil^®^ and MMC produced significant radiosensitization in HT-29 cells, with SER values of 1.4 and 1.3, respectively. However, there was no significant sensitization in SW480 cells. The antitumor efficacy was evaluated in human HT-29 and SW480 xenograft models. Animals treated with Promitil^®^ and RT (5 Gy) had significantly prolonged TGD in both tumor models compared to free MMC plus RT, demonstrating the efficacy of Promitil^®^ as a cancer therapy [38]. Promitil^®^ has finally reached clinical testing as palliative therapy for two patients with oligometastases from colorectal cancer. Both patients presented durable clinical responses to the combination of Promitil^®^ and RT, suggesting the combination as a chemoradiotherapy approach [39].

#### 2.1.3. Doxorubicin

Doxorubicin (DXR) is a tetracycline antibiotic that induces DNA damage by inhibiting topoisomerase II and generating free radicals. Another mechanism of action consists of the formation of DXR-DNA adducts and interstrand crosslinks from DXR convalently binding to DNA. This makes DXR promising to be used in combination with RT, by increasing sublethal radiation-induced damage. DXR low solubility in water and severe dose-dependent cardiotoxicity are important reasons to support its delivery in nanocarriers [45,74]. 

Liu et al. developed liposomes composed of 1,2-distearoyl-sn-glycero-3-phosphoethanolamine-N-[amino(polyethylene glycol)-2000] (DSPE-PEG2000), cholesterol (CHOL), and malate dehydrogenase (MDH) to encapsulate DXR (MLP-DXR). The hypoxic radiosensitizer 2-methyl-5-nitroimidazole-1-ethanol (metronidazole) was conjugated to hexadecanedioic acid (HA), in order to form (16-(2-(2-methyl-5-nitro-1H-imidazol-1-yl) ethoxy)-16-oxohexadecanoic acid (MHA). The MHA was then coupled with 3-dimethylaminopropane-1, 2-diol (DA) to form ester-linked MDH. The MDH lipid was used for obtaining hypoxia radiosensitizer liposomes. Liposomes without MDH were prepared as control (DLP-DXR). When tested in a xenograft glioma model obtained by intracranial injection of human glioblastoma U87 cells, MLP-DXR plus RT clearly demonstrated the strongest inhibition of glioma growth. Fourteen days after treatment, animals treated with MLP-DXR plus RT presented tumors with ~580 mm³, while those receiving RT alone and DLP-DXR plus RT presented tumors with ~4000 and ~1000 mm³, respectively [44].

DXR has been shown to be an effective therapeutic agent against malignant glioma cells. However, DXR has not been used for the treatment of brain tumors as its poor penetration across the blood–brain barrier hinders its efficacy. Liposomes encapsulating DXR have been studied to circumvent this poor penetration [42]. Labussière et al. evaluated the commercialized nonpegylated liposome of DXR (Myocet^®^) on two subcutaneous U87 and TCG4 and one intracranial U87 malignant glioma models xenografted on nude mice. After the treatment with RT alone (2 Gy), the median survival was 30.5 and 81.0 days, for U87 and TCG4 subcutaneous models, respectively. The combination of Myocet^®^ plus RT (2 Gy), increased the median survival up to 36.5 days in U87 and 93.0 days in TCG4 subcutaneous models. However, in intracranial U87 model, the median survival was 39.5 days for the RT alone and 32.0 days for the Myocet^®^ plus RT [43]. Chastagner et al. evaluated Myocet^®^ and the commercialized pegylated liposome (Caelyx^®^, DXR) on U87 xenograft model. The overall survival was 22, 47, and 48 days when mice received RT alone (2 Gy), Myocet^®^ plus RT (2 Gy) and Caelyx^®^ plus RT (2 Gy), respectively. These results showed that both Myocet^®^ and Caelyx^®^ have a synergistic interaction with RT [42]. Han et al. showed that micelles composed of polyethylene glycol (PEG), polycaprolactone (PCL), and Pluronic P105, loading DXR inhibited the drug resistance of human myelogenous leukemia (K562/ADR) cells [75]. Lung adenocarcinoma is the most common primary lung cancer, and it is often detected at the metastastic stage [76]. Therefore, Xu et al. selected the A549 cell line to evaluate micelles consisting of PEG-PCL/P105 loading DXR. CFA using A549 lung cancer cells was performed to determine the effects of the treatment with DXR-micelles plus irradiation (at 0–6 Gy). The cell survival fraction was ~0.05% and ~0.005% after treatment with irradiation alone (6 Gy) and the DXR-micelles plus RT, respectively. Moreover, SER of cells treated with DXR-micelles was 1.44 [45].

### 2.2. Natural Products as Radiosensitizers

Natural products play a role as radiosensitizers, presenting different effects on irradiated normal and tumor cells. Despite promising effects in cancer treatment, their clinical applications in RT are few. This is possibly related to their low bioavailability, which can be overcome with the use of nanocarriers [77,78,79]. 

#### Curcumin

Curcumin (CUR) is a phenylpropanoid isolated from the roots of the herbaceous perennial plant *Curcuma longa*. It is a natural antioxidant and nuclear factor (NF-κB) inhibitor, used on the treatment of inflammatory conditions and cancer and as a radiosensitizer. The radiosensitizing role of CUR arises from its interference with many different pathways. It has been shown that CUR inhibits transcription factors (NF-KB, AP-1, and STAT3) highly expressed in cancer cells, and genes involved in processes such as proliferation (COX-2, c-Myc, and cyclin D1), survival (Bcl-2, Bcl-XL), invasion (MM9), and metastasis (ICAM-1, ELAM-1, VCAM-1). It has also been reported to interfere with the cell cycle, arresting cells in the most radiosensitive G2/M phase. In this phase, cells passing the G2 checkpoint are unable to repair DNA damage. CUR has also been found to increase the intrinsic and extrinsic apoptosis pathways induced by RT [46]. 

Minafra et al. prepared solid lipid nanoparticles loaded with CUR and evaluated its sensitizing effects in breast cancer cells. CUR nanoparticles (10 µM) were combined to different doses of irradiation (2, 4, 6, and 9 Gy) in order to obtain dose-response curves. The radiosensitizing effect was determined by the dose-modifying factor (DMF) obtained from the curves and calculated at the surviving fraction of 50%. DMFs of 1.78 and 1.38 were obtained for the MCF7 cell line and the triple-negative MDA-MB-231 cell line, respectively. Shi et al. prepared liposomes encapsulating CUR (L-CUR) and evaluated its sensitizing effects in a mouse model with LL2 (murine Lewis lung carcinoma) tumor. There was a significant inhibition of tumor growth in mice treated with L-CUR plus RT (5 Gy), in relation to the animals treated with either L-CUR or RT alone (5 Gy). Twenty-two days after treatment, animals treated with L-CUR plus RT presented a tumor volume of ~300 mm^3^, while those receiving irradiation alone (6 Gy) presented a tumor volume of ~600 mm^3^ [47]. 

### 2.3. Hypoxic Cell Radiosensitizers

Hypoxia plays an important role in RT as tumor cells have been reported to be 2–3 times more radioresistant when in a hypoxic environment as compared to tumor cells under normal oxygen level [80]. This cellular response dependency on oxygen after irradiation is known as the “oxygen fixation hypothesis”. As previously mentioned, in the indirect action of ionizing radiation, ion pairs created in water react with molecules yielding free radicals (R•). These radicals cause a type of DNA base damage that can be easily repaired by antioxidants. However, peroxides (RO_2_•) formed by the reaction of molecular oxygen with the R• in DNA lead to damage that is difficult or even impossible for the cell to repair. Thus, as the hypothesis postulates, molecular oxygen can permanently fix the DNA damage caused by radicals [81,82]. Oxygen deficiency is present in the majority of solid human tumors, due to inadequate and heterogeneous vascular network. For this reason, strategies to overcome hypoxia-induced radioresistance are of clinical importance [83,84]. 

#### 2.3.1. Tirapazamine

Tirapazamine (TPZ) is a hypoxic cytotoxin, i.e., a type of radiosensitizer that is only activated under a hypoxia condition. In such a condition, TPZ is reduced into a radical intermediate (TPZ•−) which in sequence becomes protonated (TPZH•). The bioactive radicals which are precursors for DNA damage are formed in further reaction steps, still under debate. What is known is that they lead to the inactivation of the enzyme topoisomerase II, and then, DNA DSB arise. In the presence of O2, the TPZ•− radical is back-oxidized to the parent neutral state, hence the hypoxia selectivity [85,86].

Silva et al. synthetized a cupric-TPZ complex [Cu(TPZ)_2_] that improved TPZ’s hypoxia selectivity in prostate cancer, exhibited slower metabolism, and higher DNA binding, compared to TPZ [87]. Then, Silva et al. [48] developed liposomes of different lipidic compositions to load Cu(TPZ)_2._ A formulation composed of 1,2-dioleoyl-*sn*-glycero-3-phosphocholine (DOPC):CHOL:DSPE-PEG2000, when tested in C4-2B (human prostatic carcinoma cells) spheroids presented an IC50 of ~22.0 μM, significantly reduced compared to that of the free complex (~42 μM). The combination of Cu(TPZ)_2_-loaded liposomes with RT was evaluated in C4-2B spheroids, treated with 10 μM of total TPZ either as free Cu (TPZ)2 or liposomal-Cu(TPZ)_2_ for 24 h followed by X-ray irradiation at a single dose of 7 or 10 Gray. The changes in spheroids diameter were monitored for 22 days. At the end of the experiment, liposomes significantly reduced spheroid growth rate compared to RT alone or in combination with the free complex [48].

#### 2.3.2. Nitroimidazoles

Nitroimidazoles have also been studied as hypoxic cell radiosensitizers. They mimic the oxygen reacting with DNA radicals to “fix” radiation damage in hypoxic cells. The adducts formed lead to DNA strand breaks and, consequently, cell death [88]. So far, the toxicity of these compounds has limited its clinical translation making its encapsulation in nanocarriers a promising strategy [89]. Sadeghi et al. developed temperature-sensitive liposomes loaded with pimonidazole (PMZ) (TSL-PMZ). Cell survival was measured by CFA in FaDu (human hypopharyngeal carcinoma) cell line under hypoxic conditions. TSL-PMZ enhanced the effect of RT (4 Gy) in a concentration-dependent way. The SER of TSL-PMZ in concentrations of 0.25, 0.50, and 0.75 mM in FaDu cells were 1.7, 2.3, and 2.9, respectively [49]. 

### 2.4. DNA Repair Inhibitors 

Tumor cells respond to the DNA damage caused by irradiation by activating the DNA signaling response, which upregulates DNA repair. The DNA repair pathways are promising targets for therapeutic intervention such as radiosensitization [90].

#### Dbait

Dbait (DNA strand break bait) are innovative molecules composed of 32 base-pair deoxyribonucleotides. They form intramolecular DNA double helix mimicking DNA damages, acting as a bait for DNA damage signaling enzymes. This “false” DNA damage signal prevents the recruitment of repair enzymes (DNA-PK and PARP) that would act on DSB and SSB. These molecules have given promising results as radiosensitizer in several types of tumors as they inhibit several repair pathways of DNA damage induced by RT [91,92]. Yao et al. 2016 developed a polycation nanoparticle to delivery Dbait (NP-Dbait). The therapeutic efficacy of NP-Dbait was evaluated in xenograft mouse models bearing PC-3 or 22Rv1 prostate cancer. The combination of NP-Dbait and RT (9 Gy) led to significantly longer TGD and prolonged the survival time of tumor-bearing mice when compared with controls groups. When tumor volumes of control group reached 3000 mm^3^, animals treated with NP-Dbait plus RT presented tumor volumes of ~1300 mm^3 (^PC-3 model) and ~700 mm^3^ (22Rv1 model). Those receiving irradiation alone (9 Gy) presented tumor volume of ~2500 mm^3 (^PC-3 model) and ~2000 mm^3^ (22Rv1 model) [50]. 

## 3. Co-Delivery of Molecules: The Search for Synergism

The efficacy of chemotherapeutic agents is often hindered by the rapid development of drug resistance. Combination therapy based on the understanding of tumor biology, tumor environment, and molecular pathways is a successful strategy to overcome multidrug resistance [93,94]. The use of nanotechnology for cancer treatment keeps on evolving, particularly in combinatorial treatments [95,96]. The co-encapsulation of molecules in nanocarriers is a technical challenge that comes with advantages. Some of those consist of lower costs of constituents and manufacturing process as compared to two single formulations, as well as a lower adjuvant load to the patient. Another point is the uncertainty about the biodistribution when two different formulations are administered. Ultimately, established synergistic ratios can be encapsulated, guaranteeing a higher efficacy [14,15]. Nanocarriers co-encapsulating molecules are a promising strategy to increase the effectiveness of RT [95]. 

Tian et al. developed nanoparticles composed of poly(lactic-co-glycolic acid) (PLGA) coated with PEG to co-deliver paclitaxel (PTX, a chemotherapeutic drug that can cause myelosuppression and peripheral neuropathy [97]) and CDDP at a molar ratio of 1:1 (NP-PTX:CDDP 1:1). The efficacy was evaluated in murine models using both the human lung cancer 344SQ allograft and H460 xenograft models. NP-PTX:CDDP 1:1 plus RT (5 Gy) greatly retarded tumor growth rates compared to RT alone (5 Gy). Twenty-three days after treatment of mice bearing 344SQ tumors, a tumor volume variation of ~10 was observed in the animals treated with RT alone, while those receiving NP-PTX:CDDP 1:1 plus RT presented a tumor volume variation of ~5. For mice bearing H460 tumors, at the end of eighteen days, a change in tumor volume of ~12 was observed after treatment with RT alone and of ~5 after treatment with NP-PTX:CDDP 1:1 plus RT. These results showed the potential of NP-PTX:CDDP 1:1 as a promising chemoradiotherapy strategy [51]. Zhang et al. developed nanoparticles to co-encapsulate wortmannin (WTMN) and CDDP (NP-WTMN:CDDP), to enhance chemoradiotherapy and reverse platinum resistance in ovarian cancer models. These nanoparticles were evaluated in murine xenograft models of platinum-sensitive ovarian cancer (A2780 cell line) and CDDP-resistant ovarian cancer (A2780cis cell line). After treatment with NP-WTMN:CDDP followed by irradiation (5 Gy), the TGD was ~8 days in A2780 model. Moreover, the TGD was ~10 days in A2780cis model. For both models, the TGD was ~2 days, in animals treated with RT alone (5 Gy). These results showed that co-delivering WTMN:CDDP in nanoparticles is a strategy to reverse CDDP resistance and improve chemoradiotherapy efficacy [1]. In another study, Zhang et al. developed PLGA-PEG-based nanoparticles to co-deliver etoposide (ET) and CDDP at a molar ratio of 1:1.8 (NP-ET:CDDP). The efficacy of these nanoparticles in combination with RT was evaluated using both 344SQ and H460 murine lung cancer models. The TGD was ~8 days, after treatment with NP-ET:CDDP followed by 5 Gy irradiation, while for animals treated with RT alone (5 Gy), the TGD was ~2 days, for both 344SQ and H460 models [53]. 

Nanoparticles composed of PLGA-PEG conjugated to transferrin (Tf) were developed by Zhang et al. to co-deliver tetrahydrocurcumin (THC) and DXR (NP-Tf-THC:DXR) into glioma [54]. The overexpression of Tf receptors (TfR) in glioma cells is well known. Nanocarriers targeting TfR have been investigated for the effective delivery of drugs to brain tumors [98]. When tested in a xenograft glioma model obtained by subcutaneous injection of rat C6 glioma cell line, NP-Tf-THC:DXR combined with irradiation (3 Gy) showed a tumor inhibition rate of 94.49%, while that for treatment with irradiation alone (3 Gy) was ~40% [54]. 

Li et al. developed nanoparticles to co-deliver temozolomide (TMZ) and Dbait (NP-TMZ-Dbait). CFA using C6 mouse glioma cells was performed to determine the effects of the different treatments. The survival fractions of cells treated with radiation alone (3 Gy), NP-TMZ-Dbait or NP-TMZ-Dbait plus RT (3 Gy), were −0.8, ~0.60, and ~0.20, respectively, confirming the synergistic potential of the combination [55]. 

Liu et al. developed a Dbait nanoparticle to deliver docetaxel (DTX, a chemotherapeutic drug that can lead to infusion reactions, febrile neutropenia, pneumonitis, and neuropathies [99]). The combination of RT (3 Gy) and nanoparticles was evaluated in mice bearing PC-3 tumor. Thirty days after treatment, animals receiving the formulation plus RT presented a tumor volume of ~850 mm^3^. Those treated with irradiation alone (3 Gy) presented a tumor volume of ~1500 mm^3^. This was reflected on longer survival time for animals receiving both the nanoparticles and RT compared to RT alone [56]. 

Magnetic graphene oxide nanoparticles were developed by Yang et al. to encapsulate 5-fluorouracil (5-FU, a chemotherapeutic drug that can lead to mucositis, leukopenia, neutropenia, and thrombocytopenia [100]). Firstly, magnetic graphene oxide (MGO) was conjugated with PEG2000 and covalently bonded with metronidazol. These nanocomposites (NCs) were mixed with FePt magnetic nanoparticles, and 5-FU was added. CFA under 2 Gy irradiation was conducted in H1975 and A549 human lung adenocarcinoma cells, to evaluate the radiation enhancement ratio (RER) of MGO-FU:MI NCs. Survival fractions of H1975 cells changed from 0.58 with radiation alone (2 Gy) to 0.36 with combined therapy, an RER of 1.6. For A549 cells, the RER was 1.5 with survival fractions of 0.79–0.52 after treatments with radiation alone and combined therapy, respectively. The dose enhancement factor (DEF) was calculated to evaluate the effect of MGO-FU:MI NCs [57]. DEF is defined as the ratio between the dose deposited in tumor with NCs and the dose deposited in control group [101]. The DEF values were 1.68 for H1975 cells at 15 µg/mL and 1.68 for A549 cells at 10 µg/mL. These results showed the radioprotective and dose enhancement effects of MGO-FU:MI NCs. In addition, the SER values of MGO-FU:MI NCs were 1.15 for H1975 cells at 15 µg/mL and 1.99 for A549 cells at 10 µg/mL, confirming the effective role of MGO-FU:MI NCs as a radiosensitizer [57].

Hua et al. synthetized metronidazole polymers to develop nanoparticles encapsulating DXR for glioma therapy. The nanoparticles obtained [ALP-(MIs)n:DXR] were evaluated in mice bearing C6 tumor, and ALP-(MIs)n:DXR plus RT (2 Gy) had significant effects on inhibiting glioma growth compared to other treatments. Nanoparticles composed of a nonradiosensitizer polymer (PLGA) were compared to ALP-(MIs)n:DXR to further investigate the radiosensitization effect of (P-MIs)n. Both the glioma inhibition rates of ALP-(MIs)25:DXR (19.6) and ALP-(MIs)48:DXR (13.7) presented a remarkably higher inhibition efficacy toward glioma growth than the AL-PLGA:DXR (68.3), which suggests that (P-MIs)n has a radiosensitizing effect [58]. 

Liu et al. developed liposomes to deliver Dbait (MLP-Dbait) for glioma therapy. To evaluate the antitumoral efficacy of MLP-Dbait, a xenograft glioma model was obtained by intracranial injection of C6 cells to mice. Liposomes containing the green fluorescent protein (GFP) gene were used as a control group. For the combination of RT (2 Gy) with free Dbait or MLP/GFP or MLP/Dbait, the tumor growth rates were 37.9%, 39.9%, and 21.1%. This higher effectivity of the combination of Dbait, MLP, and RT was also translated to a longest median survival rate, compared to the other treatments [59]. 

Neufeld et al. developed a formulation of talazoparib and buparlisib in Hafnium and 1,4-dicarboxybenzene (Hf-BDC) nanoscale metal organic frameworks (nMOFs) coated with DSPE-PEG (TB@Hf-BDC-PEG) [60]. Hf is a high atomic metallic ion, which interacts with ionizing radiation, to generate ROS for cancer treatment [102]. The results of CFA indicated that the growth rates and colony formation abilities of 4T1 cells were significantly inhibited by RT plus TB@Hf-BDC-PEG. The RER of TB@Hf-BDC-PEG combined with irradiation (8 Gy) was 12.68. The therapeutic efficacy of TB@Hf-BDC-PEG was evaluated on Balb/C mice bearing subcutaneous 4T1 murine breast tumors. Fourteen days after treatment, there was a change in tumor volume ~6 in the animals treated with RT alone (4 Gy), while those treated with TB@Hf-BDC-PEG plus RT (4 Gy) presented a change in tumor volume of ~4.5 [60]. 

It is important to highlight that some nanocarriers themselves, such as high atomic number nanoparticles (mainly gold nanoparticles), act as radiosensitizers by enhancing radiation energy deposition in the cells. This strategy has been recently reviewed elsewhere [103,104] and is not the focus of the present review. Herein, we describe nanocarriers designed to encapsulate and deliver radiosensitizing molecules.

## 4. Nanocarriers Encapsulating Oxygen: Targeting Hypoxia

On item 2.3, we describe how hypoxia plays an important role in radioresistance and the encapsulation of hypoxic cell radiosensitizers as one strategy to overcome it. Another strategy consists of the delivery of oxygen itself using agents such as fluorocarbons (FC). FC can dissolve oxygen and then deliver it in hypoxia regions by passive diffusion [84]. Perfluorocarbons (PFCs) are chemically inert and present excellent biocompatibility. They have been extensively studied as oxygen suppliers to improve RT outcome [61]. 

Zhou et al. used perfluorotributylamine (PFTBA), a PFC with strong platelet inhibition effect to obtain PFTBA:albumin nanoparticles. These nanoparticles were stored in an oxygen chamber for PFC oxygenation. These nanoparticles were designed to function as a two-stage oxygen delivery system. Once administered, the PFTBA:albumin nanoparticles take advantage of the EPR effect to passively accumulate at the tumor and release the bound O_2_. At the same time, the PFTBA inhibits platelet activation in the tumor blood vessels disrupting the vessel barriers. This leads to higher red blood cell infiltration and consequently higher oxygen delivery to the tumor. The efficacy of combining PFTBA:albumin nanoparticles to radiation was evaluated in vivo. Balb/C mice bearing the highly hypoxic subcutaneous CT26 colon cancer tumors (~50 mm^3^) were divided in groups to receive different treatments. Fourteen days after treatment, animals treated with PFTBA:albumin nanoparticles presented tumors >700 mm³, while those receiving irradiation alone (5 Gy) presented tumors between 300 and 600 mm³. Animals receiving PFTBA:albumin nanoparticles followed 10 h later by irradiation (5 Gy) presented tumors <150 mm³. These results showed that PFTBA:albumin nanoparticles were able to sensitize the tumors to RT, effectively inhibiting tumor growth [61].

Yao et al. designed nanoparticles composed of commercial lipids and perfluoro-15-crown-5-ether (PFCE) loading CDDP prodrug (cisPt(IV)) for enhanced chemoradiotherapy efficacy. An in vivo study in mice bearing 4T1 murine breast tumor (~150 mm³ at day 0) was performed to show the hypoxic relief enhanced chemoradiotherapy. At the end of fourteen days, animals treated with PFCE@cisPt(IV)-Lip nanoparticles (at days 0 and 3) presented tumors with ~800 mm³. Animals receiving irradiation alone (6 Gy at days 1 and 4) presented tumors with ~600 mm³, and animals receiving PFCE@cisPt(IV)-Lip nanoparticles (at days 0 and 3) followed by irradiation (6 Gy at days 1 and 4) presented tumors with ~200 mm³. Tumor slices were stained with a hypoxia-probe (pimonidazole) to confirm the potency of the formulation on tumor oxygenation, as compared to a control. The semiquantitative analysis of positive hypoxia areas revealed positive area percentages of only ~2.5% for animals receiving the formulation, as compared to ~42.4% for control animals [62].

Song et al. prepared pegylated hollow Bi_2_Se_3_ nanoparticles which could be filled with perfluorohexane (a liquid PFC). This PEG-Bi_2_Se_3_ @perfluorohexane functioned as an oxygen reservoir targeting hypoxia combined with the ability of the high Z element Bi to locally concentrate the radiation energy, further sensitizing the tumor. First, it was verified in vivo that the exposure of the oxygen-loaded PEG-Bi_2_Se_3_ @perfluorohexane nanoparticles to NIR light (808 nm laser for 10 min) led to the burst release of oxygen from the nanoparticles, instantly increasing tumor oxygenation. Tumor slices stained with a hypoxia probe (pimonidazole) showed enhanced oxygenation level for tumors injected with PEG-Bi2Se3 @perfluorohexane nanoparticles and exposed to NIR light (~35%) as compared to the control (~75%). Following that, the antitumor efficacy was evaluated in Balb/c mice bearing subcutaneous 4T1 murine breast cancer tumors. Therefore, the relative tumor volume (RTV) was calculated by using the formula: RTV = Tx × 100/T0, where Tx refers to the tumor volume of the respective tumor on day x, and T0 refers to the tumor volume of the same tumor when the treatment started. Sixteen days after treatment, animals treated with oxygen-loaded PEG-Bi_2_Se_3_ @perfluorohexane nanoparticles plus NIR presented RTV of ~11. Those receiving irradiation alone (6 Gy) presented RTV of ~7.5. Animals exposed to oxygen loaded PEG-Bi_2_Se_3_ @perfluorohexane nanoparticles plus NIR plus irradiation (6 Gy) presented RTV of ~1.5. This confirmed the synergistic effect of the combination of the nanoparticles with NIR and irradiation on tumor growth suppression [63]. In another study, Song et al. prepared another multifunctional RT sensitizer. The formulation consisted of pegylated PFC nanodroplets decorated with TaOx nanoparticles (TaOx@PFC-PEG). Similarly to the previously described formulation, this is also able to deliver oxygen loaded on PFC and to concentrate radiation energy at the tumor site due to the TaOx. Again, they evaluated the antitumor efficacy in Balb/c mice bearing subcutaneous 4T1 tumors. Twelve days after treatment, animals treated with oxygen-loaded TaOx@PFC-PEG presented RTV of ~9. Those receiving irradiation alone (6 Gy) presented RTV of ~6. Treatment with oxygen-loaded TaOx@PFC-PEG plus irradiation (6 Gy) presented the highest efficacy with an RTV of ~2, confirming the radiosensitizing potential of the formulation [64]. 

Xu et al. prepared a liposomal formulation encapsulating perfluorohexane (lip-PFH). Balb/c mice bearing CT26 (mice colon cancer) tumors with ~80 mm³ were used in in vivo experiments. First, the accumulation of lip-PFH at the tumor site was verified 24 h after intravenous administration. Subsequently the antitumor efficacy was evaluated. When animals received the injection of lip(PFH) followed 24 h later by 10 Gy irradiation, the TGD was ~17.5 days. This was not that notable but significantly improved compared to the TGD (~14 days) for animals treated with irradiation only (10 Gy) [65].

## 5. Nanocarriers Designed to Boost the Abscopal Effect

The abscopal effect consists of a systemic anticancer response that can result from RT. It occurs in addition to the local effect of radiation. An antitumor response throughout the body is induced, reaching distant sites, which were not irradiated [105]. The mechanisms for the abscopal effect are not fully understood, but the immunological hypothesis supports that it occurs due to immunogenic responses initiated by RT-induced DNA breaks. This antitumor immune response, mediated by T-cell activation, leads to the regression of off-site, nonirradiated tumors [105,106]. Although considered promising, the practical efficacy of the abscopal effect is unsatisfactory. Therefore, new strategies as means to enhance it are highly desired [68]. One of these strategies consist of the development of antigen-capturing nanoparticles (AC-NPs). These nanoparticles bind to tumor antigens, released after RT-induced immunogenic cell death and improve their delivery to antigen presenting cells, enhancing cancer immunity. Antigens are then presented to T-cells, and the effector T-cells are directed to both primary and distant tumor sites boosting the abscopal effect [107,108].

Min et al. developed different poly(lactic-*co*-glycolic acid) (PLGA)-based AC-NPs, and showed that the set of captured antibodies is dependent on the surface properties of the NP. AC-NPs composed of unmodified PLGA bind to proteins through noncovalent hydrophobic–hydrophobic interactions. When coated with either amine polyethylene glycol or 1,2-dioleoyloxy-3-(trimethylammonium)propane (DOTAP), the AC-NPs bind to proteins via ionic interactions. When coated with PEG-maleimide, they bind to proteins through stable thioether bonds [66].

Yang et al. developed mesoporous silica nanoparticles (MSNs) functionalized with (3-aminopropyl) triethoxysilane (APTES) and investigated its potential to enhance the abscopal effect in the treatment of hepatocellular carcinoma. MSNs have a large surface area and porous structure that can absorb biomaterials such as antigens and deliver them to target cells. An in vivo experiment was performed in mice with bilateral Hepa1-6 (Murine hepatoma) tumors. The primary tumors received RT alone (8 Gy); MSN alone (injected into the tumor) or RT + MSN (same scheme) and the distant tumors (nonirradiated/non-treated) had their volumes evaluated. At the end of 40 days, the tumor volumes were ~1100 and ~1300 mm³ for animals treated with RT alone and MSN alone, respectively. By comparison, when RT and MSN were combined, the tumor volume was reduced to ~550 mm³ [67].

Chen et al. developed AC-NPs based on PEG-maleimide-mPEG-functionalized hollow mesoporous titanium dioxide (HTiO_2_) aimed at eradicating metastatic breast tumor. In this formulation, the PEG-maleimide is responsible for capturing the tumor-associated antigens. To further boost the abscopal effect, this formulation was used to encapsulate an indole-amine-2,3-dioxygenase inhibitor (IDOi). IDO is an immunosupressive cytosolic enzyme often overexpressed in the tumor. The potential of the formulation on activating the abscopal effect was investigated in mice with bilateral 4T1 (murine mammary carcinoma) tumors. Animals received different formulations and irradiation (4 Gy) on the primary tumor, and the RTV of distant tumors (non-irradiated) was evaluated. At the end of 20 days, the RTV was ~4.5 and ~3.0 for animals treated with RT alone and HTiO_2_-mPEG, respectively. By comparison, adding PEG-maleimide to the formulation (HTiO_2_- mal-mPEG) allowed for an RTV of ~1.0 and combining PEG-maleimide and an IDOi to the formulation (IDOi@HTiO_2_- mal-mPEG) led to an RTV of ~0.5. This study made it clear that the combination of RT with immunotherapy provided by the formulation was highly effective on tumor suppression [68].

## 6. Radiation-Triggered Delivery Systems

Nanocarriers can be designed to be triggered when exposed to endogenous or exogenous stimuli at the tumor site. This prevents drug release in the bloodstream, sparing normal tissues and allowing maximum antitumor efficacy [7,109]. X-rays, as other exogenous triggering stimuli, have the advantages of being delivered to a precise region and on demand. As a main limitation, X-rays do not access and treat the metastatic sites of uncertain location [110]. The high clinical translation potential of X-rays is due to its high tissue penetration and the fact that it is already used to treat tumors [69]. The capacity of bond breaking and ROS generation have been explored as means of triggering delivery systems.

The first demonstration of X-ray triggered release from a nanocarrier leading to higher cytotoxicity was published by Starkewolf et al. in 2013 [69]. The nanocarrier consisted of DNA strands attached to gold nanoparticles (AuNP). DXR can be loaded by conjugation to the DNA strands [69,111]. When irradiated with X-rays, the generated ROS break the DNA strands, triggering the release of the DXR. CFA using MCF-7 (human breast cancer) cells was performed to determine the effects of different treatments. When not irradiated, DXR–DNA–AuNP (0.80 cell survivability) and DXR alone (0.81 cell survivability) presented similar toxicity and were more toxic than DNA–AuNP (0.92 cell survivability). Upon 5 Gy irradiation, DXR–DNA–AuNP (0.20 cell survivability) were more toxic than radiation alone (0.32 cell survivability) DXR alone (0.28 cell survivability) and DNA–AuNP (0.30 cell survivability). The extra toxicity of DXR–DNA–AuNP was attributed to the triggering irradiation [69].

Zhang et al. developed bismuth nanoparticles functionalized with S-nitrosothiol (Bi-SNO NPs). When irradiated, the X-ray breaks the S-N bond, triggering the release of large amounts of nitric oxide (NO). NO is a gas signal molecule of vasodilation and can therefore alleviate tumoral hypoxia. The efficacy of this formulation was evaluated in vivo in mice bearing subcutaneous U14 (murine cervical carcinoma) tumors (70 mm^3^). At the end of 14 days, animals receiving RT alone (5 Gy) and Bi-SNO NPs presented RTV of ~12.5 and ~14, respectively. The combination of RT (5 Gy) and Bi-SNO NPs led to synergistic activity, and RTV was significantly reduced (~2) [70].

Combining the strategies of co-delivery and X-ray triggering, Fan et al. developed pegylated thioether-hybridized hollow mesoporous organosilica nanoparticles (HMONs) encapsulating tert-butyl hydroperoxide (TBHP) and iron pentacarbonyl (Fe(CO)_5_). X-ray irradiation of the formulation activates a cascade release of •OH and CO. Upon irradiation, a peroxy bond cleavage takes place within TBHP, generating the highly oxidative •OH radical, for enhanced RT. This radical attacks the Fe(CO)_5_ releasing CO for gas therapy, leading to mitochondria exhaustion followed by cell apoptosis. The formulation was evaluated in vivo for its efficacy in mice bearing subcutaneous U87MG glioma tumor (~6–8 mm). Animals received either irradiation alone (8 Gy) or different formulations followed by irradiation (8 Gy) 24 h later. Twenty days after the treatments, the RTV was similar (~5.5) for animals receiving either irradiation alone or HMON-Fe(CO)_5_ followed by irradiation. For animals receiving HMON-TBPH followed by irradiation, the RTV was ~4.5. Treatment with the co-loaded formulation (HMON-TBPH-Fe(CO)_5_) followed by irradiation was able to cause tumor regression with an RTV close to zero. When administered without irradiation, the HMON-TBPH-Fe(CO)_5_ formulation was not able to suppress tumor growth (RTV ~9.5) as compared to a PBS control (RTV ~10.5), confirming the synergistic efficacy of co-encapsulated compounds and irradiation [71]. 

Deng et al. designed liposomes triggered by X-ray irradiation. In this work, a liposomal formulation was embedded with a photosensitizer (verteporfin) and gold nanoparticles. Irradiation induces ^1^O_2_ generation by the photosensitizer. Singlet oxygen acts by oxidizing unsaturated lipids, leading to liposomal membrane destabilization and cargo release. The gold nanoparticles interact with X-rays acting as a radiosensitizer. These liposomes were used to encapsulate DXR and evaluated for its activity in xenograft mouse model bearing HCT 116 colorectal cancer. Animals were divided into groups to receive PBS, liposome only, irradiation only (4 Gy), or liposome plus irradiation (4 Gy), and the ability of these treatments to control tumor growth was evaluated. Two weeks post-treatment, the tumor volume increase was verified for animals receiving PBS (3.0-fold), liposome only (2.9-fold), and irradiation only (3.4-fold). On the other hand, treatment with liposome plus irradiation allowed for tumor size reduction (74%) as compared to the PBS control group [72]. 

Zu et al. developed liposomes encapsulating hemoglobin (Hb) and DXR. In this formulation, Hb alleviates hypoxia, and DXR release is radio-triggered by the action of ROS in the lipid membrane. When evaluated for ROS generation, this was higher in B16 (murine melanoma) tumors after treatment with the formulation plus irradiation as compared to either formulation alone or irradiation alone. Regarding the triggered release of DXR, 10 min after 2 Gy irradiation, up to 6% of content was released. When the irradiation dose was enhanced to 8 Gy, up to 20% of DXR was released. For the nonirradiated formulation, only ~1% of DXR was released in the same time. As expected, when evaluated for antitumor efficacy in a highly radioresistant tumor model (C57BL/6 mice bearing B16), RT alone (8 Gy) was not able to significantly inhibit tumor growth. Combining RT to the formulation co-encapsulating Hb and DXR allowed for significant tumor growth inhibition. This was superior to the combination of RT plus liposomes encapsulating Hb only or RT plus liposomes encapsulating DXR only, confirming the synergistic effects of the combination [73].

Two liposomal formulations designed to be irradiation-triggered were patented by Fologea et al. One formulation consists of pH-sensitive liposomes designed to encapsulate an agent and an organic halogen (such as chloral hydrate, fluoral hydrate, or bromal hydrate). The triggering mechanism relies on the reaction of the organic halogen with water in the vesicle’s aqueous interior upon irradiation. This reaction releases H+, leading to a drop in the internal pH and disruption of the pH-sensitive bilayer, releasing the encapsulated content [112]. The other liposomal formulation was designed to contain a nanoscintillator responsive to irradiation, an enzyme capable of hydrolyzing the liposomal membrane, and an enzyme activator in a photolabile molecular cage. Triggered release occurs in a series of events that take place upon irradiation. When the nanoscintillator is irradiated, light is emitted, and the photolabile molecular cage with an enzyme activator is disrupted. The enzyme is then activated to disrupt the liposomal membrane releasing the encapsulated agent [113]. Another patent by Bondurant et al. relates to liposomes composed of a stable forming lipid, an ionizing radiation polymerizable colipid, and a chain transfer agent. Upon irradiation, radical species produced in water can initiate radical chain polymerizations in the polymerizable colipid (e.g., polymerizable phosphatidylcholines), leading to membrane destabilization and content release. The transfer of irradiation-generated free radical ions by the chain transfer agent (such as thiols or esters) increases the polymerization of the radiation polymerizable colipid, enhancing the trigger capacity [114].

## 7. Microbeam and Minibeam Radiation Therapy to Modulate the EPR Effect

**Figure 3:**
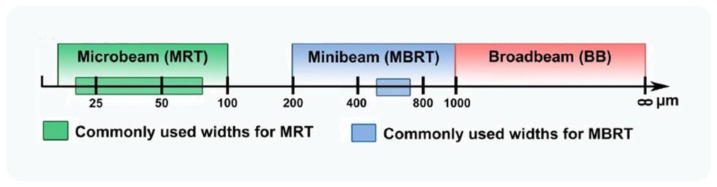
Schematic representation of commonly used widths for microbeam, minibeam, and broadbeam radiotherapy preclinical studies. Reproduced from Brönnimann et al., Nature Publishing Group, 2016 [115].

Aiming at the reduction of radiation-induced side effects, many novel RT treatment strategies are arising. Spatially fractionated radiotherapy such as microbeam radiation therapy (MRT) and minibeam radiation therapy (MBRT) have attracted interest in the last years. Figure 3 represents commonly used beam widths for MRT and MBRT.

### 7.1. Microbeam Radiation Therapy

MRT was proposed by Slatkin and coworkers in 1992 [116]. In MRT, a multislit collimator is used in order to shape an X-ray beam so that the dose deposition profile consists of areas of peaks and valleys, spatially modulated on the micrometer scale (beam width ranging from 25 to 100 μm). On the peak areas, doses might consist of several hundred Grays, while valley regions present doses below the level of tissue tolerance. This distinct dose deposition enables a substantial reduction on toxicity to the normal tissue combined with a tumor control rate similar to that observed with conventional RT. The increased therapeutic index that can be obtained with MRT is of high importance for improved cancer treatment with radiation [117,118]. The mechanisms that underlie the increased antitumor efficacy combined with the sparing of surrounding normal tissues are not yet fully understood. One of the suggested biological mechanisms consists of the fact that MRT damages preferentially the tumor microvasculature in comparison to normal tissue microvasculature. It has been demonstrated that normal tissue mature vessels are highly tolerant against peak (high) doses going through transiently increased permeability only, while immature tumoral vessels undergo complete disruption [119,120,121].

With this in mind, MRT can be used to modulate the EPR effect. It is true that the EPR effect has been validated in both experimental animal models and different human tumors [3]. However, the importance and extent of the EPR effect in patients is still the focus of intense debate. The EPR effect is known to be more pronounced in small animal models, usually used to evaluate the preclinical efficacy of nanocarriers, as compared to human tumors. This is one explanation for the disbalance between the high amount of promising preclinical nanocarriers and the relative small number of these carriers in the clinical setting [122]. Therefore, a strategy to enhance tumor vessel permeability optimizing the EPR effect is highly desirable. 

Sabatasso et al. demonstrated in mice bearing U-87 human glioblastoma that tumor irradiation with MRT (synchrotron generated parallel X-rays in a range of 25–50 µm in width) led to FITC-dextran (~27 nm) extravasation in this area. In nonirradiated animals, administered FITC-dextran remained intravascular with no extravasation to the control tumors. When animals were treated with MRT (150 Gy peak dose), a twofold increase in FITC-dextran transpermeability was observed 45 min postirradiation, as compared to the control. A study with FluoSpheres^TM^ (100 nm microspheres) showed that they remained constrained to MRT paths. This indicates, as expected, a size-dependent transpermeability following MRT. It was possible to conclude from this study that the transient vascular permeability induced by MRT can be a strategy to improve the delivery of nanocarriers to the tumor site [123]. 

### 7.2. Minibeam Radiation Therapy 

Despite promising preclinical studies for MRT, its production depends on large synchrotron facilities, of which there are currently only a few around the world. In addition to the difficulty to access these facilities, specialized hospitals are rarely close to them, hindering the clinical application of MRT [124]. In order to overcome this problem, efforts are put into using nonsynchrotron irradiators (such as a conventional X-ray tube) to produce microbeams [125]. Another strategy consists of using slightly wider ‘minibeams’ (beam widths ~500–700 μm), which are more easily produced with custom multislit collimators placed in small animal irradiators, as compared to microbeams [124,126,127]. Minibeam radiation therapy (MBRT) leads to similar radiobiological effects as compared to MRT and is also hypothesized to improve the delivery of nanocarriers. This idea was evaluated by Price et al. on a study of the effects of MBRT on the delivery of liposomal DXR. An in vivo study was performed on mice with syngeneic orthotopic triple-negative breast cancer. Animals were irradiated with either MBRT (28 Gy) or broadbeam radiation therapy (7 Gy) and 24 h later received the liposomal DXR. The delivery of liposomal DXR to the tumor was increased by 7.1-fold for MBRT and 2.7-fold for broadbeam radiation therapy compared to liposomal DXR alone (sham RT) [127]. 

## 8. Limitations and Future Directions

Besides the clear advantages of combining nanocarrier-assisted delivery of molecules and RT, which were presented in this review, there are also limitations that must be considered. Among limitations that come from the nanocarriers, it is important to remember that none of them are completely safe and nontoxic. This is affected by the lack of knowledge regarding their distribution and action, controlled by characteristics such as size, charge, degradation, and cancer stage and type, among others [128,129]. The commercial viability and availability of nanocarriers also have to be considered. Some of the most important challenges are: Possible scale-up problems, low production rate, difficulties in separating by-products, lack of quality control system, inconsistencies in reproducibility, and the high cost [130]. These limitations help us understand why the impact of nanocarrier-assisted drug delivery on human patients and clinical outcome has so far been insufficient [131]. For their future wide application, collaborative research efforts with more in vivo studies and clinical trials are necessary [128]. If the developed products are novel, safe, and meet the medical needs with cost effective benefits, the investment in nanocarrier development is likely to increase [130].

RT, in turn, has as its major limitation the damage caused to normal surrounding tissues. In this manner, modern RT and imaging techniques are enhancing the applications of radiation oncology. To the best of our knowledge, the nanocarrier-assisted delivery of molecules has only been evaluated in combination with photon RT, as reviewed herein. Photon RT is also the treatment modality applied to most patients worldwide. However, particle therapy, such as proton and carbon ions, is a promising strategy to be evaluated in combination with nanocarrier drug delivery. These therapy modalities are characterized by steep dose falloff with a minimal exit dose beyond a specified target. This allows for a more accurate and precise treatment, reducing healthy tissue exposure and, therefore, the toxicity [132]. Nevertheless, it is important to have in mind that such techniques have a high cost. So far, their use is concentrated in high-income countries that have centers able to deliver these particles [133]. 

In addition, the combination of nanocarrier drug delivery and RT has only been evaluated with single RT courses. Fractionated and hypofractionated RT should be evaluated in future in vivo studies [132].

Another important point is the heterogeneity in the RT response observed for tumors of the same histology [134]. Response prediction by means of liquid biopsy and circulating biomarkers allows for a refined treatment with patient stratification by biological parameters. Molecularly designed systems to target specific cancer will lead to future personalized cancer care [132,135].

## 9. Conclusions

In this review, we present the state of the art of nanocarriers designed to be used in combination with RT. So far, these systems were used to encapsulate one of or a combination of different radiosensitizing molecules such as classic chemotherapeutic drugs, natural products, hypoxic cell radiosensitizers, or DNA repair inhibitors. Nanocarriers have also been successfully designed to encapsulate and deliver oxygen, modulating the hypoxic TME; or to capture and deliver antibodies to antigen-presenting cells, boosting the abscopal effect. X-ray irradiation was shown to be an efficient exogenous trigger to nanoparticles and liposomes, enhancing the therapeutic ratio of these formulations. Finally, we presented how the transient and efficient vascular permeability arising from spatially fractionated RT modalities, such as microbeam and minibeam radiation therapy, can modulate the EPR effect and enhance the passive delivery of nanocarriers to the tumor site. All these different strategies of combining nanocarrier-assisted delivery of molecules and RT were validated in in vitro and/or in vivo studies. Thus, nanotechnology can be used to overcome the limitations of RT and the other way around. 

## Abbreviations

A2780	platinum-sensitive murine ovarian cancer
A549	human non-small-cell lung cancer cell
C4-2B	human prostatic carcinoma cell
C6	rat glioma cell line
CDDP	cisplatin
CT26	colon carcinoma cell line
CUR	curcumin
DTX	docetaxel
DXR	doxorubicin
ET	etoposide
FaDu	human hypopharyngeal carcinoma cell
HCT 116	human colorectal carcinoma cell line
HT-29	human colorectal adenocarcinoma cell
H1975	human non-small-cell lung cancer cell
H460	human non-small-cell lung cancer cell
K562/ADR	human myelogenous leukemia
LL2	murine Lewis lung carcinoma
MCF-7	human breast cancer cell line with estrogen, progesterone and glucocorticoid receptors
MDA-MB-231	triple-negative breast cancer cell
MMC	mitomycin C
PC3	human prostate adenocarcinoma cell
PMZ	pimonidazole
PTX	paclitaxel
TCG4	human glioma cell line
U14	murine cervical carcinoma
THC	tetahydrocurcumin
TMZ	temozolomide
TPZ	tirapazamine
U87	human primary glioblastoma cell
WTMN	wortmannin
SW480	human colon adenocarcinoma cell
22Rv1	human prostate carcinoma cell
344SQ	human lung cancer cell
4T1	murine mammary carcinoma cell line
5-FU	5-fluorouracil
